# Methanol Poisoning Leading To Brain Death: a Case Report

**DOI:** 10.2478/jccm-2021-0039

**Published:** 2021-11-13

**Authors:** Jakub Glowala, Jeremy B. Richards

**Affiliations:** 1 McGaw Medical Center of Northwestern University, Chicago, IL, USA; 2 Beth Israel Deaconess Medical Center, Boston, MA, USA

**Keywords:** methanol toxicity, brain death

## Abstract

**Introduction:**

The COVID-19 pandemic has put increased stress on medical systems, infrastructure, and the public in expected and unexpected ways. This case report summarises an unexpected case of methanol poisoning from hand sanitiser ingestion due to changes in industry regulations, increased demand for cleaning products and severe psychosocial stressors brought on by the pandemic. Severe methanol toxicity results in profound metabolic disturbances, damage to the retina and optic nerves, and potentially death.

**Case Presentation:**

The patient was a 26-year-old male with alcohol use disorder who presented with one day of nausea, vomiting, and abdominal pain after consuming hand sanitiser. Within a few hours, the patient had suffered multiple seizures, cardiac arrests and required admission to the ICU for emergent management of methanol poisoning. EEG and brain perfusion imaging were performed to confirm brain death, given concerns about the cranial nerve exam after methanol poisoning.

**Conclusions:**

While rare, methanol toxicity remains a potentially fatal poisoning in the United States and worldwide. When healthcare and public resources are strained, healthcare professionals must consider particularly abnormal presentations. In patients suspected of brain death from methanol toxicity, cranial nerve examination may be unreliable. Therefore, additional testing is necessary to confirm brain death.

## Introduction

The COVID-19 pandemic has transformed the modern world, and many of the indirect harms to patients will only become evident in retrospect. For patients with alcohol use disorder, the pandemic has increased alcohol-seeking behaviours and alcohol consumption and increased withdrawal rates due to decreased access to ethanol [1]. In addition, with the increased use of harsh cleaning agents, strained production lines potentially vulnerable to contamination, and messaging advocating consumption of potentially lethal substances, patients may be particularly susceptible to poisoning [2].

This case describes an atypical source of methanol toxicity in a young male with alcohol use disorder. It highlights the need for a pathophysiologic approach to clinical medicine during times of uncertainty. It also emphasises the need to avoid anchoring even if there is a known source of poisoning and highlights the difficulty of interpreting neurologic examinations in the setting of methanol toxicity.

## Case Description

A 26-year-old male with alcohol use disorder was brought to Emerson Hospital, Concord, MA, USA, in the early morning for evaluation. The patient suffered from nausea, vomiting, and abdominal pain after drinking multiple bottles of hand sanitiser on the previous day. On arrival, he was confused and appeared unwell but was able to confirm he had ingested hand sanitiser and no other substances.

In the Emergency Department, he rapidly worsened, becoming unresponsive and suffered multiple seizures. He then suffered five brief cardiac arrests, with the return of spontaneous circulation following administration of epinephrine and bicarbonate, all within the first few hours of presentation.

He was intubated after this final cardiac arrest, approximately 30 minutes after becoming unresponsive; he had a Glasgow Coma Scale (GCS) of 3, accompanied by seizures and several cardiac arrests. A head and abdominal computerised tomography scan were undertaken immediately but showed no particular abnormalities.

Laboratory studies (Table 1) demonstrated a severe anion gap metabolic acidosis and hyperosmolality. He was initiated on norepinephrine (0.5mg/mL administered at 0.5mcg/kg/min IV [Hospira, Inc., Lake Forest, IL, USA]), phenylephrine (20mcg/mL administered at 3mcg/kg/min IV [Eton Pharmaceuticals, Deer Park, IL, USA]), vasopressin (20 units/mL administered at 2.4 units/hr IV [Par Pharmaceutical, Chestnut Ridge, NY, USA]), and epinephrine (4mcg/L administered at 0.1mcg/kg/min IV [Par Pharmaceutical, Chestnut Ridge, NY, USA]).

**Table 1:** Summary of patient’s lab values on arrival to the ED and upon completion of dialysis.

	On Arrival	Post-Dialysis
WBC	17.5	12.3
Hgb	12.8	10.0
Hct	42.6	31.6
Plt Count	248	82
Glucose	83	144
Urea	13	2
Creatinine	1.9	0.7
Na	150	150
K	6.4	4.2
Cl	114	116
HCO3	<2	25
Anion Gap	Unmeasurable	9
Calcium	7.5	7.8
Phos	8.8	4.1
Mg	3.7	1.9
Osmolality	427	299
Ven pO2	182	129
Ven pCO2	44	36
Ven pH	6.53	7.49
Lactate	19.0	1.5
ALT	85	245
AST	99	1192
Creatine Kinase		31470
Lipase	75	
cTroponinT	<0.01	<0.01

Over the following few hours, five litres of intravenous fluids were administered for persistently low blood pressures and mean arterial pressures, monitored with an arterial catheter placed at the time of intubation.

He was initiated on fomepizole (1gm/mL administered at 15 mg/kg, followed by 10 mg/kg every 12 hours for 4 doses, then 15 mg/kg every 12 hours IV (Jazz Pharmaceuticals, Inc. Palo Alto, CA, USA).

Due to the concern about the possibility of non-ethanol alcohol toxicity, despite the patient’s lack of access to any other poisons at home and denial of any other ingestions, the night of his presentation, he was transferred to an academic hospital, Beth Israel Deaconess Medical Center, Boston, USA.

On arrival, the patient’s GCS was 3. He had a heart rate of 80bpm, blood pressure 94/52mmHg on the four vasopressors described above; an oxygen saturation of 97% on volume assist-control ventilation with a tidal volume of 440cc, respiratory rate of 34 breaths per minute, positive end-expiratory pressure of 8cm H_2_O, and a fraction of inspired oxygen of 50%. His pupils were 3mm in diameter with no response to light. He was comatose, with no response to noxious stimuli. On the recommendation of the nephrology service, given the presence of severe acidosis, a bicarbonate infusion was started.

Fomepizole (Jazz Pharmaceuticals, Inc. Palo Alto, CA, USA) was continued, and thiamine (100mg/mL, 500mg IV [FFF Enterprises, Temecula, CA, USA], folate (1mg tablet, 1mg PO [LGM Pharma, Boca Raton, FL, USA]), and pyridoxine (100mg/mL, 100mg, IV [Biocare SD, Tempe, AZ, USA]) were provided, given concern for toxic alcohol ingestion.

The nephrology service was consulted early in the morning of Day 1 post-admission and he was admitted to the medical intensive care unit; emergency dialysis was commenced.

Targeted temperature management (TTM) was initially deferred given the severity of his condition.

His alcohol panel was positive for methanol but otherwise negative. In addition, his osmolar gap was serially monitored, given the relatively long processing time of methanol testing.

The morning after admission, his pH was 7.3 with continuous renal replacement therapy (CRRT) and a continuous bicarbonate infusion. Targeted temperature management was initiated at that time and maintained for 24 hours. Continuous renal replacement therapy was discontinued once his osmolar gap closed from an initial 118.0 mOsm/kg to -10 mOsm/kg (normal range -14.0 to 10.0).

Moreover, his methanol levels were undetectable. His metabolic derangements resolved after 24 hours on dialysis, he no longer required vasopressors, and his temperature was recorded at 37^0^ Celsius.

**Fig.1:**
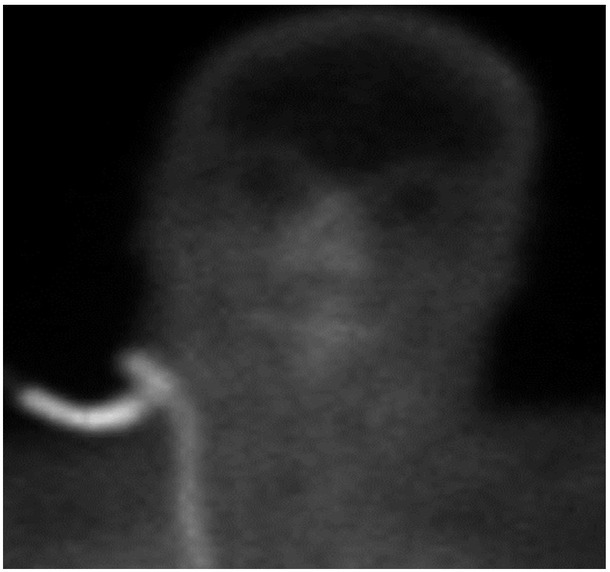
Brain perfusion scan demonstrating no discernible uptake by the brain.

On Day 3, post-admission, a neurologic exam was performed. Brain stem reflexes were absent; repeat head CT demonstrated diffuse oedema consistent with global hypoxia and ischemia. An electroencephalogram did not demonstrate cerebral activity, and apnea testing was consistent with brain death. Despite all examination manoeuvres indicating brain death, we could not rule out that optic nerve damage from methanol toxicity was potentially obfuscating the cranial nerve exam, so a brain perfusion scan (Figure 1) was performed. This confirmed brain death.

## Discussion

Methanol poisoning is relatively rare and potentially fatal toxicity in the United States and throughout the world. Roughly 90% of cases are unintentional ingestions, and the most common sources (84.5%) are automotive products [3,4]. Though most exposures are ingestions, methanol can be toxic through oral, pulmonary, or skin contact [5]. Hand sanitiser, though not meant to be ingested, may be used by patients as a means of intoxication when ethanol is unavailable. Given the health risks associated with methanol, hand sanitisers are prohibited explicitly from containing this compound. However, given the COVID pandemic, there has been both a massive influx of new hand sanitiser manufacturers as well as the loosening of Federal Drug Administration (FDA) manufacturing oversight [6].

**Fig. 2:**
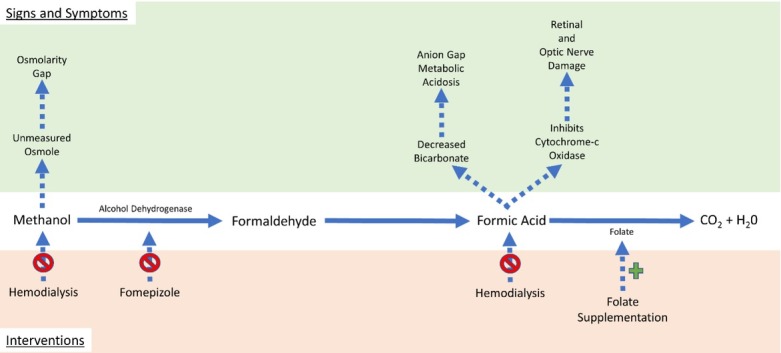
Graphical summary of the methanol pathway with the resulting signs and interventions.

Given the rarity of severe presentations and the unexpected sources of exposure, this case describes key features of methanol toxicity. Methanol is a toxic alcohol that is an unmeasured osmol, elevating the osmolar gap [7]. It is metabolised to formaldehyde and then, via aldehyde dehydrogenase, to formic acid. The latter is a toxic metabolite and causes an anion-gap metabolic acidosis [7]. As a potent alcohol dehydrogenase inhibitor, fomepizole inhibits the formation of formic acid, and dialysis is used to remove methanol [8,9]. In addition, despite no randomised controlled trials supporting its use, folate is often provided to augment formic acid elimination, given its role as a cofactor in the formic acid oxidation pathway (Figure 2) [10].

Upon resolution of acute intoxication and confirmation of clearance of serum methanol, patients should be evaluated for neurologic damage, particularly blindness due to optic nerve atrophy and retinal damage [11]. In this case, the patient’s severe acidosis and multiple cardiac arrests led to anoxic brain injury. Given potential retinal and optic nerve damage, it was unclear if the lack of corneal reflex was a feature of brain death or optic nerve death from methanol toxicity. Given the unreliable neurologic exam, brain perfusion imaging was used to confirm brain death.

**Fig. 3:**
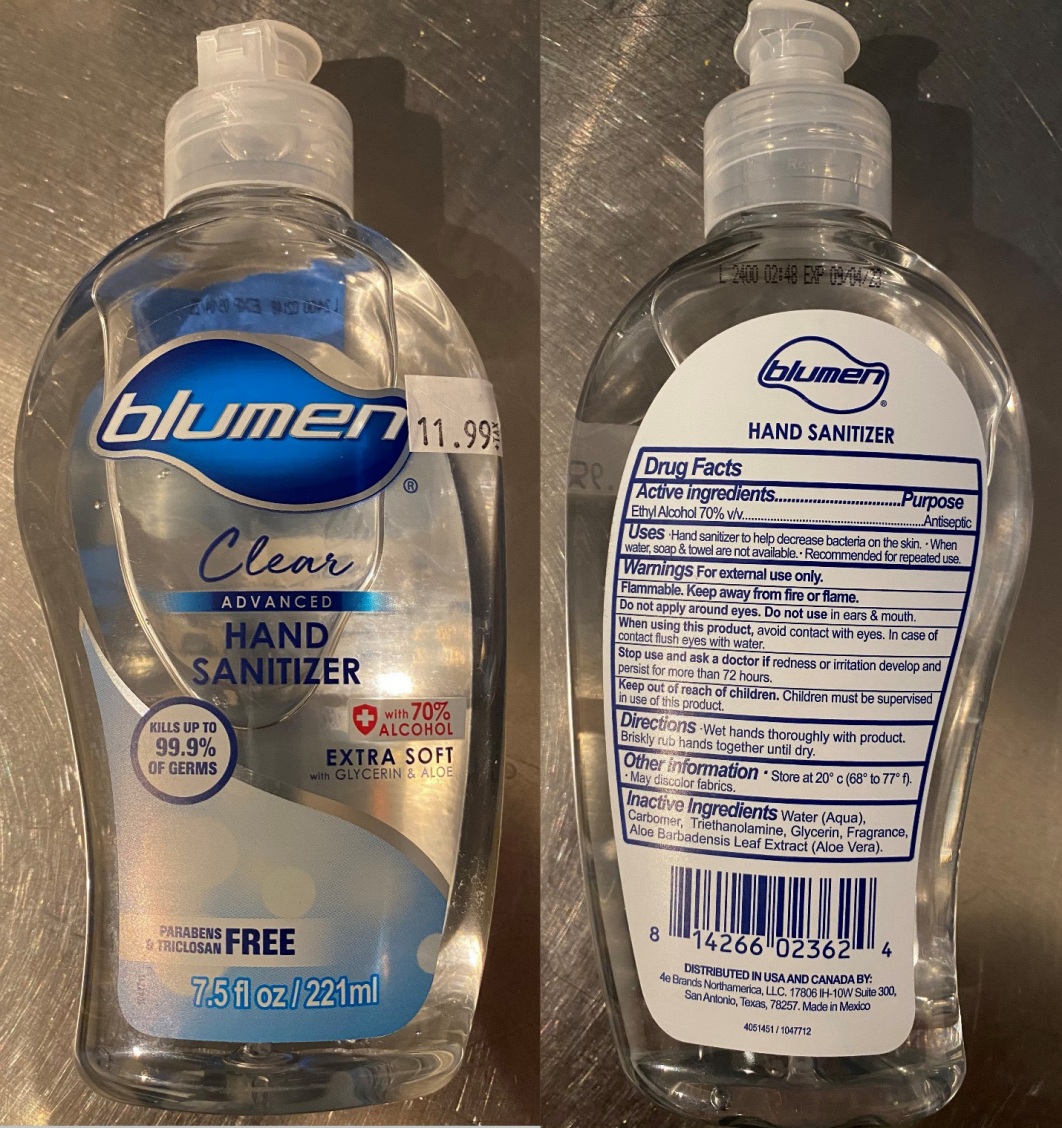
Image of hand sanitiser confirmed to contain methanol.

After his death, the Federal Drug Administration issued a press release about methanol-contaminated hand sanitisers, including confirmation that the product consumed by the patient (Figure 3) contained methanol. This brand of hand sanitiser was subsequently removed from the market [12]. This highlights a key clinical principle as the significant number of contaminated hand sanitisers was unknown at the time of the patient’s presentation. Furthermore, the patient had clearly ingested hand sanitiser without other possible sources of ingestion. A diagnosis could have been delayed without understanding the first principles and careful analysis of the patient’s metabolic derangements. It is crucial to maintain a broad differential as toxic exposures or ingestions can come from unexpected sources. In addition, capacity strain in the setting of a global viral pandemic increases the risk of health care providers missing esoteric or unexpected diagnoses and requires even further vigilance on the part of clinicians.

An additional diagnostic dilemma demonstrated by this case is the difficulty and absolute necessity of having a clear, definitive diagnosis of brain death. While in this case, the physical exam and brain perfusion imaging were congruent, not having recognised that this patient’s neurologic exam was affected by the mechanism of death could result in misdiagnosis of brain death in a patient with methanol toxicity. As such, health care providers must be aware that brain death cannot be confirmed until the patient is hemodynamically stable and all evidence of metabolic derangement is corrected, and no potential sources of false-negative results exist. In an unreliable examination, such as in this patient, ancillary testing, such as EEG and brain perfusion magnetic resonance imaging (MRI), is crucial to prevent the misdiagnosis of brain death.

## Conclusion

In summary, even when exposure history is unclear or seemingly incompatible with methanol poisoning, a broad differential must be considered. Elevated serum osmolarity and metabolic acidosis should raise concerns for non-ethanol alcohol toxicity. Empiric treatment can be started with fomepizole, folate supplementation, and hemodialysis while confirmation laboratory test results are pending. Nevertheless, severe toxicity and delay in the presentation can result in neurologic damage or death. In such cases, patients may be unresponsive, precluding attempts to assess visual acuity. Given that the diagnosis of brain death is contingent on a reliable physical exam, retinal and optic nerve damage should be assumed, and corroborating evidence through EEG and brain perfusion imaging, along with expert neurologic consultation, must be performed before declaring a patient brain dead.
